# Laughable

**Published:** 1848-10

**Authors:** 


*Laughable.*—The editor of one of our most agreeable contemporaries, recently noticed our mistake, in stating that *five* instead of *six* months was recommended for the lecture term, in the *report* submitted at the last meeting of the American Association, and read us this lesson: "Editors should be very careful how they make statements, which, when incorrect, can be so easily and completely refuted." We shall say nothing of the moral tone of this remark, but merely for the amusement of our readers quote what the same editor said in his journal just one month before:

"The last action of the American Medical Association was to reduce the lecture term from six to five months. To this change several colleges, we suspect a majority of the better organized ones, had already cheerfully acceded, throughout the country. The term is probably sufficient for the present; it is as long as young gentlemen just now can be made to remain patient, and to receive any really useful information from the lips of their teachers."

Truly, the editor is blessed with a short memory.

